# Chronic rhinosinusitis increases the risk of hemorrhagic and ischemic stroke: A longitudinal follow-up study using a national sample cohort

**DOI:** 10.1371/journal.pone.0193886

**Published:** 2018-03-01

**Authors:** Woo Hyun Lee, Jeong-Whun Kim, Jae-Sung Lim, Il Gyu Kong, Hyo Geun Choi

**Affiliations:** 1 Departments of Otolaryngology, Kangwon National University Hospital, Chuncheon, Korea; 2 Departments of Otorhinolaryngology, Seoul National University College of Medicine, Seoul National University Bundang Hospital, Seongnam, Korea; 3 Department of Neurology, Hallym University Sacred Heart Hospital, Anyang, Korea; 4 Departments of Otorhinolaryngology-Head & Neck Surgery, Hallym University College of Medicine, Anyang, Korea; National Yang-Ming University Hospital, TAIWAN

## Abstract

**Background:**

Several studies have reported that chronic rhinosinusitis (CRS) increases the risk of stroke. The aim of this study is to elucidate the putative association between CRS and stroke (ischemic or hemorrhagic) using large population-based national health insurance data.

**Methods:**

Using the national cohort study from the Korean Health Insurance Review and Assessment Service, CRS patients (n = 22,959) and control participants (n = 91,836) were selected and matched at a rate of 1:4 (age, sex, income, region, hypertension, diabetes, and dyslipidemia). A Cox-proportional hazard model was used to analyze the hazard ratio (HR) of CRS for hemorrhagic stroke and ischemic stroke. We divided the participants according to age and gender for the subgroup analysis.

**Results:**

The HR for hemorrhagic and ischemic stroke was significantly increased in the CRS patients compared to that in the controls (adjusted HR = 2.43, 95% confidence interval [CI] = 2.10–2.80 for hemorrhagic stroke; adjusted HR = 1.76, 95% CI = 1.61–1.92 for ischemic stroke) after adjusting for age, sex, income, region of residence, hypertension, diabetes, dyslipidemia, ischemic heart disease, migraine, chronic kidney disease, depression, sleep disorder, and chronic obstructive pulmonary disorder. In the subgroup analysis, the HR of hemorrhagic stroke was significantly increased in the CRS group regardless of age and gender. The HR of ischemic stroke was also significantly increased in all subgroups of the CRS group.

**Conclusion:**

CRS consistently increased the risk of ischemic and hemorrhagic stroke regardless of age and gender.

## Introduction

Chronic rhinosinusitis (CRS) is a prevalent disease defined as inflammation of the nose and the paranasal sinuses lasting more than 12 weeks [1]. Patients with CRS present with symptoms of nasal blockage, rhinorrhea, post-nasal drip, facial pain, or smell disorders. The GA^2^LEN study reported an overall prevalence of CRS of 10.9% in the European population, [2], while the investigated prevalence of CRS was 14% in the U.S.A [3]. In Korea, 6.2% of the general population was diagnosed with CRS according to a questionnaire and physical examination [4]. CRS is a common chronic disease and is associated with a socioeconomic burden. Rhinosinusitis may lead to life-threatening intracranial complications, such as subdural empyema, cavernous sinus thrombophlebitis, meningitis, and brain abscess [5].

Stroke is an abrupt interruption in cerebral blood flow causing brain cell death followed by neurological deficits. Ischemic and hemorrhagic stroke are the two major stroke types. The modifiable risk factors of stroke include hypertension, smoking, high waist-to-hip ratio, physical inactivity, hyperlipidemia, diabetes mellitus, and alcohol consumption [6].

Recently, several studies based on large population insurance data indicated a close relationship between CRS and stroke [7,8]. The suggested hypotheses for this association were anatomical proximity of the sinus and brain, inflammation-mediated emboli or spasm of cerebral arteries, adverse reactions to an associated allergy medicine, or complications following sinus surgery [7–10].

The aim of this study is to elucidate the putative association between CRS and stroke using large population-based national health insurance data. We identified this association for both hemorrhagic and ischemic stroke.

## Methods

### Study population and data collection

The ethics committee of Hallym University (2014-I148) approved the use of these data. Written informed consent was exempted by the Institutional Review Board.

This national cohort study relies on data from the Korean Health Insurance Review and Assessment Service-National Sample Cohort (HIRA-NSC). The Korean National Health Insurance Service (NHIS) selects samples directly from the entire population database to prevent non-sampling errors. Approximately 2% of the samples (one million) were selected from the entire Korean population (50 million). These selected data were classified at 1,476 levels (age [18 categories], sex [2 categories], and income level [41 categories]) using randomized stratified systematic sampling methods via proportional allocation to represent the entire population. After data selection, the appropriateness of the sample was verified by a statistician who compared the data from the entire Korean population to the sample data. The details of the methods used to perform these procedures are provided by the National Health Insurance Sharing Service [11]. This cohort database includes (i) personal information, (ii) health insurance claim codes (procedures and prescriptions), (iii) diagnostic codes using the International Classification of Disease-10 (ICD-10), (iv) death records from the Korean National Statistical Office (using the Korean Standard Classification of disease), (v) socioeconomic data (residence and income), and (vi) medical examination data for each participant over the period ranging from 2002 to 2013.

Because all Korean citizens are recognized by a 13-digit resident registration number from birth to death, exact population statistics can be determined using this database. It is mandatory for all Koreans to enroll in the NHIS. All Korean hospitals and clinics use the 13-digit resident registration number to register individual patients in the medical insurance system. Therefore, the risk of overlapping medical records is minimal, even if a patient moves from one place to another. Moreover, all medical treatments in Korea can be tracked without exception using the HIRA system. In Korea, notice of death to an administrative entity is legally required before a funeral can be held. The causes and date of death are recorded by medical doctors on a death certificate.

### Participant selection

Of 1,125,691 cases with 114,369,638 medical claim codes, we included only participants who were diagnosed with chronic sinusitis (ICD-10: J32). Among them, we selected the participants who were treated ≥ 2 times, those who underwent head and neck CT evaluations (Claim codes: HA401-HA416, HA441-HA443, HA451-HA453, HA461-HA463, or HA471-HA473), and those not diagnosed with nasal polyps (J33) (n = 34,572). The participants were followed up to 12 years.

The history of admission for hemorrhagic stroke (I60: Subarachnoid hemorrhage, I61: Intracerebral hemorrhage, and I62: Other nontraumatic intracranial hemorrhage) and ischemic stroke (I63: Cerebral infarction) were identified using ICD-10 codes. We selected participants who were treated ≥ 1 time. These methods were used in other studies to evaluate the incidence of stoke in Korea [12,13].

**Fig 1:**
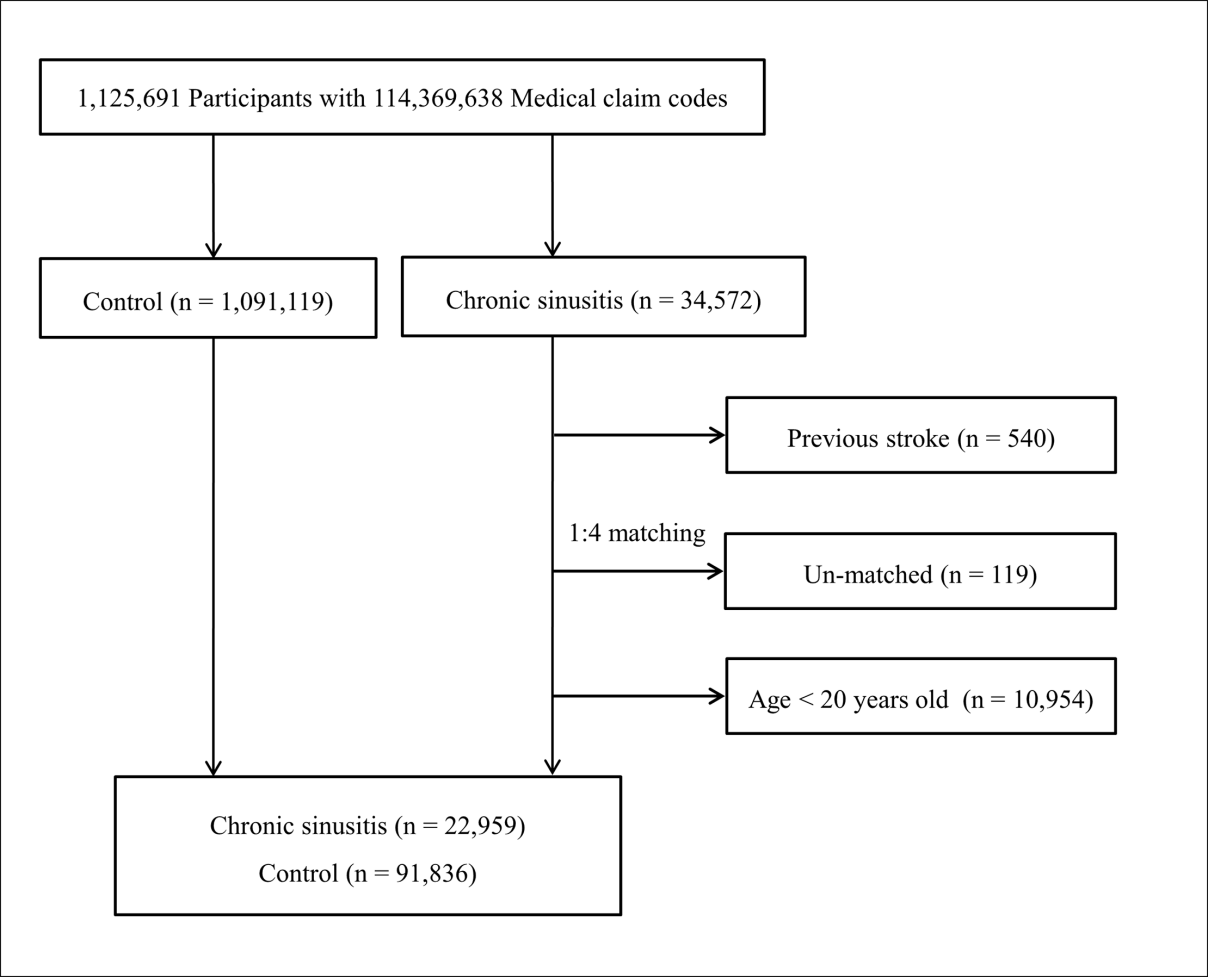
A schematic illustration of the participant selection process that was used in the present study. Of 1,125,691 cases with 114,369,638 medical claim codes, 34,572 CRS participants were selected. The participants of previous stroke history were excluded (n = 540). The CRS participants were matched 1:4 with a control group who had not been diagnosed with CRS. Un-matched CRS participants (n = 119) and less than 20 years old participants (n = 10,954) were excluded. Finally, 22,959 CRS participants and 91,836 control participants were included.

CRS patients were matched at a ratio of 1:4 with participants (control group) who had not been diagnosed with CRS from 2002 through 2013. The participants were matched for age group, sex, income group, region of residence, and past medical history (hypertension, diabetes, and dyslipidemia). To prevent selection bias when selecting the matched participants, the control group participants were sorted by a random order. It was assumed that the matched control participants were involved at the same time as each matched CRS participant (index date). Therefore, participants in the control group who died before the index date were excluded. In both the CRS and control groups, participants who had histories of hemorrhagic or ischemic stroke before the index date were excluded. In the chronic sinusitis group, 540 participants were excluded. CRS patients for whom we could not identify sufficient matching participants were excluded (n = 119). We excluded participants under 20 years old (n = 10,954). Finally, 1:4 matching resulted in the inclusion of 22,959 CRS patients and 91,836 control participants (Fig 1). However, they were not matched for ischemic heart disease, migraine, chronic kidney disease, depression, sleep disorder, and chronic obstructive pulmonary disorder histories in that strict matching increases the drop out the participant due to lack of control participants.

### Variables

Age groups were classified using 5-year intervals: 20–24, 25–29, 30–34…, and 85+ years. A total of 14 age groups were designated. The income groups were initially divided into 41 classes (one health aid class, 20 self-employment health insurance classes, and 20 employment health insurance classes). These groups were re-categorized into 11 classes (class 1 [lowest income]-11 [highest income]). Region of residence was divided into 16 areas according to administrative district. These regions were regrouped into urban (Seoul, Busan, Daegu, Incheon, Gwangju, Daejeon, and Ulsan) and rural areas (Gyeonggi, Gangwon, Chungcheongbuk, Chungcheongnam, Jeollabuk, Jeollanam, Gyeongsangbuk, Gyeongsangnam, and Jeju).

The past medical histories of participants were evaluated using ICD-10 codes. For the accuracy of diagnosis, hypertension (I10 and I15), diabetes (E10-E14), dyslipidemia (E78), migraine (G43), and chronic kidney disease (N18) were evaluated for participants who were treated ≥ 2 times. Ischemic heart disease (I20 and I25) were check if the participants were treated ≥ 1 time. Depression was defined F31 (bipolar affective disorder) through F39 (unspecified mood disorder) who treated ≥ 2 times by a psychiatrist. Sleep disorder was defined G47 who treated ≥ 2 times. Chronic obstructive pulmonary disease (COPD) was defined following the previous study [14].

### Statistical analysis

A chi-square test was used to compare the general characteristics between the CRS and control groups.

A Cox-proportional hazard model was used to calculate the hazard ratio (HR) of CRS for stroke (hemorrhagic or ischemic). In these analyses, crude (simple) and adjusted (age, sex, income, region of residence, hypertension, diabetes, dyslipidemia, ischemic heart disease, migraine, chronic kidney disease, depression, sleep disorder, and chronic obstructive pulmonary disorder) models were used. We calculated the HR and 95% confidence interval (CI). For the subgroup analysis, we divided the participants by age and sex (20–39 years old, 40–59 years old, 60+ years old; men and women). We performed other subgroup analyses according to follow up periods (≤ 1 year, ≤ 3 years, and ≤ 5 years).

Two-tailed analyses were conducted, and *P* values less than 0.05 were considered to indicate statistical significance. The results were statistically analyzed using SPSS v. 21.0 (IBM, Armonk, NY, USA).

## Results

Mean interval from index date through outcome (hemorrhagic stroke) was 50.7 months ± 35.1 in CRS group and 51.4 ± 35.4 months in control. In ischemic stroke, it was 48.8 ± 35.1 months in CRS group and 49.9 ± 34.8 months in control. The general characteristics of the control group were exactly matched with those of the CRS group. However, the prevalence of stroke (hemorrhagic or ischemic), ischemic heart disease, migraine, chronic kidney disease, depression, sleep disorder, and chronic obstructive pulmonary disorder were significantly increased in the CRS group (*P* < 0.001) (Table 1).

**Table 1:** General characteristics of participants.

Characteristics		Total participants		
		CRS (n, %)	Control (n, %)	P-value
Age (years old)				1.000
	20–24	1,329 (5.8)	5,316 (5.8)	
	25–29	1,770 (7.7)	7,080 (7.7)	
	30–34	2,200 (9.6)	8,800 (9.6)	
	35–39	2,332 (10.2)	9,328 (10.2)	
	40–44	2,318 (10.1)	9,272 (10.1)	
	45–49	2,474 (10.8)	9,896 (10.8)	
	50–54	2,526 (11.0)	10,104 (11.0)	
	55–59	2,257 (9.8)	9,028 (9.8)	
	60–64	1,894 (8.2)	7,576 (8.2)	
	65–69	1,705 (7.4)	6,820 (7.4)	
	70–74	1,165 (5.1)	4,660 (5.1)	
	75–79	654 (2.8)	2,616 (2.8)	
	80–84	248 (1.1)	992 (1.1)	
	85+	87 (0.4)	348 (0.4)	
Sex				1.000
	Male	9,589 (41.8)	38,356 (41.8)	
	Female	13,370 (58.2)	53,480 (58.2)	
Income				1.000
	1 (lowest)	383 (1.7)	1,532 (1.7)	
	2	1,383 (6.0)	5,532 (6.0)	
	3	1,465 (6.4)	5,860 (6.4)	
	4	1,508 (6.6)	6,032 (6.6)	
	5	1,669 (7.3)	6,676 (7.3)	
	6	1,868 (8.1)	7,472 (8.1)	
	7	2,067 (9.0)	8,268 (9.0)	
	8	2,443 (10.6)	9,772 (10.6)	
	9	2,825 (12.3)	11,300 (12.3)	
	10	3,228 (14.1)	12,912 (14.1)	
	11 (highest)	4,120 (17.9)	16,480 (17.9)	
Region of residence				1.000
	Urban	11,300 (49.2)	45,200 (49.2)	
	Rural	11,659 (50.8)	46,636 (50.8)	
Hypertension				1.000
	Yes	8,648 (37.7)	34,592 (37.7)	
	No	14,311 (62.3)	57,244 (62.3)	
Diabetes				1.000
	Yes	4,796 (20.9)	19,184 (20.9)	
	No	18,163 (79.1)	72,652 (79.1)	
Dyslipidemia				1.000
	Yes	7,465 (32.5)	29,860 (32.5)	
	No	15,494 (67.5)	61,976 (67.5)	
Ischemic heart disease				<0.001*
	Yes	1,855 (8.1)	5,458 (5.9)	
	No	21,104 (91.9)	86,378 (94.1)	
Migraine				<0.001*
	Yes	3,271 (14.2)	5,835 (6.4)	
	No	19,688 (85.8)	86,001 (93.6)	
Chronic kidney disease				<0.001*
	Yes	320 (1.4)	1,024 (1.1)	
	No	22,639 (98.6)	90,812 (98.9)	
Depression				<0.001*
	Yes	3,790 (16.5)	8,304 (9.0)	
	No	19,169 (83.5)	83,532 (91.)	
COPD				<0.001*
	Yes	2,673 (11.6)	5,867 (6.4)	
	No	20,286 (88.4)	85,969 (93.6)	
Sleep disorder				<0.001*
	Yes	2,418 (10.5)	5,215 (5.7)	
	No	20,541 (89.5)	86,621 (94.3)	
Hemorrhagic stroke				<0.001*
	Yes	312 (1.4)	517 (0.6)	
	No	22,647 (98.6)	91,319 (99.4)	
Ischemic stroke				<0.001*
	Yes	756 (3.3)	1,712 (1.9)	
	No	22,203 (96.7)	90,124 (98.1)	

COPD: chronic obstructive pulmonary disorder

*Chi-square test or Fisher’s exact test. Significance at P < 0.05

In the Cox-proportional hazard regression model, the crude HRs of hemorrhagic stroke and ischemic stroke were 2.43 (95% CI = 2.11–2.79) and 1.78 (95% CI = 1.64–1.94), respectively, in the CRS group, which were statistically significant. After adjusting for age, sex, income, region of residence, hypertension, diabetes, dyslipidemia, ischemic heart disease, migraine, chronic kidney disease, depression, sleep disorder, and chronic obstructive pulmonary disorder, the HRs for both subtypes of stroke were still significantly increased in the CRS group (adjusted HR = 2.43, 95% CI = 2.10–2.80 for hemorrhagic stroke; adjusted HR = 1.76, 95% CI = 1.61–1.92 for ischemic stroke) (Table 2).

**Table 2:** Crude and adjusted hazard ratios (95% confidence interval) of chronic sinusitis for hemorrhagic stroke and ischemic stroke.

Characteristics		Hemorrhagic stroke				Ischemic stroke			
		Crude	P-value	Adjusted†	P-value	Crude	P-value	Adjusted†	P-value
Chronic sinusitis			<0.001*		<0.001*		<0.001*		<0.001*
	Yes	2.43 (2.11–2.79)		2.43 (2.10–2.80)		1.78 (1.64–1.94)		1.76 (1.61–1.92)	
	No	1.00		1.00		1.00		1.00	

* Cox-proportional hazard regression model, Significance at P < 0.05

† Adjusted model for age, sex, income, region of residence, hypertension, diabetes, dyslipidemia, ischemic heart disease, migraine, chronic kidney disease, depression, sleep disorder, and chronic obstructive pulmonary disorder histories

In the subgroup analysis, the HR of hemorrhagic stroke was significantly increased in the CRS group regardless of age and sex. For example, middle-aged (40–59 years) men with CRS had a 3.23-fold (crude HR = 3.20) increased risk of hemorrhagic stroke compared to the control group (95% CI = 2.32–4.49, *P* < 0.001). The HR of ischemic stroke was also significantly increased in all subgroups of the CRS group (Table 3).

**Table 3:** Subgroup analysis of crude and adjusted hazard ratios (95% confidence interval) of chronic sinusitis for hemorrhagic stroke and ischemic stroke according to age and sex.

Characteristics		Hemorrhagic stroke				Ischemic stroke			
		Crude	P-value	Adjusted†	P-value	Crude	P-value	Adjusted†	P-value
**Young men (20–39 years old, n = 16,560)**									
Chronic sinusitis			0.013*		0.077		<0.001*		<0.001*
	Yes	2.23 (1.18–4.19)		1.81 (0.94–3.51)		2.97 (1.66–5.29)		2.89 (1.60–5.22)	
	No	1.00		1.00		1.00		1.00	
**Young women (20–39 years old, n = 21,595)**									
Chronic sinusitis			0.005*		0.005*		<0.001*		0.003*
	Yes	2.47 (1.32–4.60)		2.49 (1.32–4.71)		3.59 (1.87–6.90)		2.83 (1.43–5.60)	
	No	1.00		1.00		1.00		1.00	
**Middle aged men (40–59 years old, n = 19,715)**									
Chronic sinusitis			<0.001*		<0.001*		<0.001*		<0.001*
	Yes	3.20 (2.32–4.42)		3.23 (2.32–4.49)		2.25 (1.81–2.80)		2.19 (1.75–2.73)	
	No	1.00		1.00		1.00		1.00	
**Middle aged women (40–59 years old, n = 28,160)**									
Chronic sinusitis			<0.001*		<0.001*		<0.001*		<0.001*
	Yes	3.06 (2.24–4.17)		2.96 (2.15–4.07)		1.88 (1.47–2.42)		1.82 (1.41–2.36)	
	No	1.00		1.00		1.00		1.00	
**Old men (60+ years old, n = 11,670)**									
Chronic sinusitis			<0.001*		<0.001*		<0.001*		<0.001*
	Yes	1.99 (1.48–2.68)		1.98 (1.46–2.68)		1.59 (1.36–1.85)		1.58 (1.35–1.85)	
	No	1.00		1.00		1.00		1.00	
**Old women (60+ years old, n = 17,095)**									
Chronic sinusitis			<0.001*		<0.001*		<0.001*		<0.001*
	Yes	2.05 (1.56–2.69)		2.13 (1.61–2.81)		1.69 (1.47–1.95)		1.66 (1.43–1.91)	
	No	1.00		1.00		1.00		1.00	

* Cox-proportional hazard regression model, Significance at P < 0.05

† Adjusted model for age, sex, income, region of residence, hypertension, diabetes, dyslipidemia, ischemic heart disease, migraine, chronic kidney disease, depression, sleep disorder, and chronic obstructive pulmonary disorder histories

The adjusted HR of hemorrhagic stroke was 2.00 (95% CI = 1.36–2.95) within 1 year and 2.24 (95% CI = 1.87–2.69) within 5 years. For ischemic stroke, the adjusted HR was 1.50 (95% CI = 1.20–1.88) within 1 year and 1.75 (95% CI = 1.57–1.95) within 5 years of follow-up (each of *P* < 0.001, Table 4).

**Table 4:** Subgroup analysis of crude and adjusted hazard ratios (95% confidence interval) of chronic sinusitis for hemorrhagic stroke and ischemic stroke according to follow up periods.

Characteristics		Hemorrhagic stroke				Ischemic stroke			
		Crude	P-value	Adjusted†	P-value	Crude	P-value	Adjusted†	P-value
**Within 1 year**									
Chronic sinusitis			<0.001*		<0.001*		<0.001*		<0.001*
	Yes	2.08 (1.42–3.03)		2.00 (1.36–2.95)		1.50 (1.20–1.87)		1.50 (1.20–1.88)	
	No	1.00		1.00		1.00		1.00	
**Within 3 years**									
Chronic sinusitis			<0.001*		<0.001*		<0.001*		<0.001*
	Yes	2.47 (2.00–3.05)		2.45 (1.96–3.04)		1.70 (1.49–1.93)		1.68 (1.47–1.92)	
	No	1.00		1.00		1.00		1.00	
**Within 5 years**									
Chronic sinusitis			<0.001*		<0.001*		<0.001*		<0.001*
	Yes	2.29 (1.91–2.73)		2.24 (1.87–2.69)		1.77 (1.59–1.97)		1.75 (1.57–1.95)	
	No	1.00		1.00		1.00		1.00	

* Cox-proportional hazard regression model, Significance at P < 0.05

† Adjusted model for age, sex, income, region of residence, hypertension, diabetes, dyslipidemia, ischemic heart disease, migraine, chronic kidney disease, depression, sleep disorder, and chronic obstructive pulmonary disorder histories

## Discussion

CRS increased the risk of hemorrhagic and ischemic stroke in the present study. In the subgroup analyses according to age and sex, the risk of stoke was consistently increased in CRS participants.

Most CRS cases and complications are usually mild and self-limiting. However, cerebral complications (subdural empyema, meningitis, cavernous sinus thrombophlebitis, and brain abscess) caused by CRS can be associated with severe sequelae, as they can be disabling in 25% of cases and even lead to death in 10% of cases [15,16]. Mechanisms of cerebral complications following CRS have been suggested. The direct invasion of the CRS pathogen through congenital or acquired bony destruction could be one possible pathway. Hematogenous spread (valveless veins or arterial emboli) might be another possible pathway for cerebral complications of CRS [17]. However, sufficient evidence to support the association between CRS and stroke is lacking.

Several case reports have suggested that stroke is related to sinusitis [10,18,19]. Based on this putative association, two nationwide population-based studies recently reported the association between sinusitis and stroke. Wu et al. found that patients with CRS have a 1.39-fold increased risk of stroke compared to controls during a 3-year follow-up period [7]. In our study, the CRS patients had a 2.43-fold increased risk of hemorrhagic stroke and a 1.76-fold increased risk of ischemic stroke compared to the control group. These consistent results support the association between CRS and stroke. Kang et al. reported that during a 5-year follow-up period, ischemic and unspecified stroke were significantly more prevalent in CRS patients. However, no difference was observed in the prevalence of intracerebral hemorrhage in CRS patients [8]. This is different from our results. Our study showed that the risk of hemorrhagic stroke was also significantly increased in CRS patients. This discrepancy may be due to differences in follow-up periods. The progression of CRS is slow and gradual, and a long time is likely necessary to observe intracranial complications such as stroke. Our study had a longer follow-up period than the other study (10 years vs 5 years).

In the subgroup analysis, the risk of stroke showed a definitive relationship with CRS in all groups. The incidence of stroke increases with age and is higher in females after 55 years of age [20]. However, our results showed that CRS patients have a higher likelihood of stroke regardless of age or gender. The HR of stroke was smaller in 60+ year old group compare to that of in <40 and 40–59 years old group (Table 3). Old age may render increased exposure to risk factors of stroke such as cardiovascular problems. At young ages, CRS may be an important risk factor for the development of stroke. Moreover, the HR of stroke was increased over time following CRS and was significantly increased within 1 year (Table 4). This finding supports the causal relationship of the development of stroke following CRS. Considering these findings, early and aggressive CRS treatment may prevent stroke and its accompanying complications.

There are several hypotheses to explain the causal relationship between CRS and stroke. The sphenoid and posterior ethmoid sinus share anatomical proximity with the internal carotid artery. The adjacent sinus and internal carotid artery are separated by an approximately 0.1-mm thin bony wall, and carotid artery bulging into the sinus was found in 8% of patients with CRS [21]. Infectious aneurysms resulting from distal vessel wall involvement of pathogen may cause intracranial hemorrhage or cerebral infarction [22].

The inflammatory cytokines of CRS, such as interleukin-1 and C-reactive protein, are also speculated to play a role in the development of stroke [23,24]. Interleukin-1 may result in perivascular inflammation and progress to thrombosis of the internal carotid artery [18]. Elevated C-reactive protein levels were associated with ischemic stroke in a meta-analysis [25].

In many previous epidemiologic studies, CRS showed a strong association with cigarette smoking [26,27]. The mucociliary clearance of the sinuses is disturbed by tobacco smoke, which subsequently increases inflammation [28]. Cigarette smoking is a major risk factor for stroke that exhibits a dose-response relationship and contributes to a stroke mortality rate of approximately 15% [29,30]. The hazardous effects of cigarette smoking may explain the relationship between CRS and stroke.

Oral decongestant is commonly used to relieve symptoms of nasal obstruction in CRS. It increases the systolic blood pressure and heart rate, which may increase the risk of stroke [31]. As an example, from 2002 to 2004, the exposure rate of phenylpropanolamine which is associated with hemorrhagic stroke was 0.6% to 1.6% among hospital visiting patients in Korea [32]. Intracranial hemorrhage is a rare cerebral complication after functional endoscopic sinus surgery that is performed to treat CRS. The treatment modalities for alleviating the symptoms of CRS could be causes of stroke, though their contributions were not precisely assessed.

Our study has an advantage over previous nation-wide studies. First, the duration of the follow-up period was 3 to 5 years in previous large population-based studies. We followed the study population for 10 years, which would allow sufficient time for the progression of stroke following CRS. In addition, we performed subgroup analysis according to follow-up duration. Second, the control group was matched not only for the basic characteristics of the CRS group but also for risk factors of stroke, such as hypertension, diabetes and dyslipidemia. This detailed matching might provide valid evidence for the effect of CRS on stroke.

This study has several limitations. Although this study used a representative large population and was matched and adjusted for possible confounders, other confounders were still present, such as cardiovascular comorbidities, emotional disorders, smoking, alcohol intake, and drug usage. In addition, the degree of sinusitis was not considered in the present study. Similarly, the durations and severities of sinusitis were not consistent among the study population. In addition, missed diagnoses could not be excluded. Only CRS patients who were diagnosed by clinicians were included in this study. We did not examine the medication histories of participants.

## Conclusion

In conclusion, CRS consistently increases the risk of ischemic and hemorrhagic stroke regardless of age and gender.

## References

[1] Fokkens WJ, Lund VJ, Mullol J, Bachert C, Alobid I, Baroody F, et al (2012) EPOS 2012: European position paper on rhinosinusitis and nasal polyps 2012. A summary for otorhinolaryngologists. Rhinology 50: 1–12. doi: 10.4193/Rhino50E2 22469599 10.4193/Rhino12.000

[2] Hastan D, Fokkens WJ, Bachert C, Newson RB, Bislimovska J, Bockelbrink A, et al (2011) Chronic rhinosinusitis in Europe—an underestimated disease. A GA(2)LEN study. Allergy 66: 1216–1223. doi: 10.1111/j.1398-9995.2011.02646.x 21605125 10.1111/j.1398-9995.2011.02646.x

[3] Pleis JR, Lethbridge-Cejku M (2006) Summary health statistics for U.S. adults: National Health Interview Survey, 2005. Vital Health Stat 10: 1–153. 17252928

[4] Lee WH, Hong SN, Kim HJ, Ahn S, Rhee CS, Lee CH, et al (2015) Effects of cigarette smoking on rhinologic diseases: Korean National Health and Nutrition Examination Survey 2008–2011. Int Forum Allergy Rhinol 5: 937–943. doi: 10.1002/alr.21553 26034006 10.1002/alr.21553

[5] Bayonne E, Kania R, Tran P, Huy B, Herman P (2009) Intracranial complications of rhinosinusitis. A review, typical imaging data and algorithm of management. Rhinology 47: 59–65. 19382497

[6] O'Donnell MJ, Xavier D, Liu L, Zhang H, Chin SL, Rao-Melacini P, et al (2010) Risk factors for ischaemic and intracerebral haemorrhagic stroke in 22 countries (the INTERSTROKE study): a case-control study. Lancet 376: 112–123. doi: 10.1016/S0140-6736(10)60834-3 20561675 10.1016/S0140-6736(10)60834-3

[7] Wu CW, Chao PZ, Hao WR, Liou TH, Lin HW (2012) Risk of stroke among patients with rhinosinusitis: a population-based study in Taiwan. Am J Rhinol Allergy 26: 278–282. doi: 10.2500/ajra.2012.26.3783 22801014 10.2500/ajra.2012.26.3783

[8] Kang JH, Wu CS, Keller JJ, Lin HC (2013) Chronic rhinosinusitis increased the risk of stroke: a 5-year follow-up study. Laryngoscope 123: 835–840. doi: 10.1002/lary.23829 23377773 10.1002/lary.23829

[9] Mayberg MR (1998) Cerebral vasospasm. Neurosurg Clin N Am 9: 615–627. 9668192

[10] Rochat P, von Buchwald C, Wagner A (2001) Sinusitis and ischemic stroke. Rhinology 39: 173–175. 11721511

[11] The National Health Insurance Sharing Service of Korea.

[12] Hong KS, Bang OY, Kang DW, Yu KH, Bae HJ, Lee JS, et al (2013) Stroke statistics in Korea: part I. Epidemiology and risk factors: a report from the korean stroke society and clinical research center for stroke. J Stroke 15: 2–20. doi: 10.5853/jos.2013.15.1.2 24324935 10.5853/jos.2013.15.1.2 PMC3779679

[13] Kim RB, Kim BG, Kim YM, Seo JW, Lim YS, Kim HS, et al (2013) Trends in the incidence of hospitalized acute myocardial infarction and stroke in Korea, 2006–2010. J Korean Med Sci 28: 16–24. doi: 10.3346/jkms.2013.28.1.16 23341707 10.3346/jkms.2013.28.1.16 PMC3546096

[14] Kim J, Rhee CK, Yoo KH, Kim YS, Lee SW, Park YB, et al (2013) The health care burden of high grade chronic obstructive pulmonary disease in Korea: analysis of the Korean Health Insurance Review and Assessment Service data. Int J Chron Obstruct Pulmon Dis 8: 561–568. doi: 10.2147/COPD.S48577 24277985 10.2147/COPD.S48577 PMC3838475

[15] Giannoni CM, Stewart MG, Alford EL (1997) Intracranial complications of sinusitis. Laryngoscope 107: 863–867. 9217120 10.1097/00005537-199707000-00005

[16] Giannoni C, Sulek M, Friedman EM (1998) Intracranial complications of sinusitis: a pediatric series. Am J Rhinol 12: 173–178. 9653474 10.2500/105065898781390127

[17] Fokkens W, Lund V, Mullol J, European Position Paper on R, Nasal Polyps g (2007) European position paper on rhinosinusitis and nasal polyps 2007. Rhinol Suppl 20: 1–136. 17844873

[18] Perez Barreto M, Sahai S, Ameriso S, Ahmadi J, Rice D, et al (2000) Sinusitis and carotid artery stroke. Ann Otol Rhinol Laryngol 109: 227–230. doi: 10.1177/000348940010900220 10685578 10.1177/000348940010900220

[19] Righini CA, Bing F, Bessou P, Boubagra K, Reyt E (2009) An acute ischemic stroke secondary to sphenoid sinusitis. Ear Nose Throat J 88: E23–28. 19924653

[20] Roger VL, Go AS, Lloyd-Jones DM, Benjamin EJ, Berry JD, Borden WB, et al (2012) Heart disease and stroke statistics—2012 update: a report from the American Heart Association. Circulation 125: e2–e220. doi: 10.1161/CIR.0b013e31823ac046 22179539 10.1161/CIR.0b013e31823ac046 PMC4440543

[21] Arslan H, Aydinlioglu A, Bozkurt M, Egeli E (1999) Anatomic variations of the paranasal sinuses: CT examination for endoscopic sinus surgery. Auris Nasus Larynx 26: 39–48. 10077255 10.1016/s0385-8146(98)00024-8

[22] Hurst RW, Judkins A, Bolger W, Chu A, Loevner LA (2001) Mycotic aneurysm and cerebral infarction resulting from fungal sinusitis: imaging and pathologic correlation. AJNR Am J Neuroradiol 22: 858–863. 11337328 PMC8174942

[23] Tokushige E, Itoh K, Ushikai M, Katahira S, Fukuda K (1994) Localization of IL-1 beta mRNA and cell adhesion molecules in the maxillary sinus mucosa of patients with chronic sinusitis. Laryngoscope 104: 1245–1250. 7523818 10.1288/00005537-199410000-00011

[24] Yildirim YS, Apuhan T, Kocoglu E, Simsek T, Kazaz H (2011) High sensitivity C-reactive protein levels in chronic rhinosinusitis and allergic rhinitis. Kulak Burun Bogaz Ihtis Derg 21: 266–269. doi: 10.5606/kbbihtisas.2011.039 21919832 10.5606/kbbihtisas.2011.039

[25] Emerging Risk Factors Collaboration, Danesh J, Erqou S, Walker M, Thompson SG, Tipping R, et al (2007) The Emerging Risk Factors Collaboration: analysis of individual data on lipid, inflammatory and other markers in over 1.1 million participants in 104 prospective studies of cardiovascular diseases. Eur J Epidemiol 22: 839–869. doi: 10.1007/s10654-007-9165-7 17876711 10.1007/s10654-007-9165-7

[26] Lieu JE, Feinstein AR (2000) Confirmations and surprises in the association of tobacco use with sinusitis. Arch Otolaryngol Head Neck Surg 126: 940–946. 10922224 10.1001/archotol.126.8.940

[27] Chen Y, Dales R, Lin M (2003) The epidemiology of chronic rhinosinusitis in Canadians. Laryngoscope 113: 1199–1205. doi: 10.1097/00005537-200307000-00016 12838019 10.1097/00005537-200307000-00016

[28] Benninger MS, Ferguson BJ, Hadley JA, Hamilos DL, Jacobs M, Kennedy DW, et al (2003) Adult chronic rhinosinusitis: definitions, diagnosis, epidemiology, and pathophysiology. Otolaryngol Head Neck Surg 129: S1–32. 12958561 10.1016/s0194-5998(03)01397-4

[29] Thun MJ, Apicella LF, Henley SJ (2000) Smoking vs other risk factors as the cause of smoking-attributable deaths: confounding in the courtroom. JAMA 284: 706–712. 10927778 10.1001/jama.284.6.706

[30] Bhat VM, Cole JW, Sorkin JD, Wozniak MA, Malarcher AM, Giles WH, et al (2008) Dose-response relationship between cigarette smoking and risk of ischemic stroke in young women. Stroke 39: 2439–2443. doi: 10.1161/STROKEAHA.107.510073 18703815 10.1161/STROKEAHA.107.510073 PMC3564048

[31] Salerno SM, Jackson JL, Berbano EP (2005) Effect of oral pseudoephedrine on blood pressure and heart rate: a meta-analysis. Arch Intern Med 165: 1686–1694. doi: 10.1001/archinte.165.15.1686 16087815 10.1001/archinte.165.15.1686

[32] Yoon BW, Bae HJ, Hong KS, Lee SM, Park BJ, Yu KH, et al (2007) Phenylpropanolamine contained in cold remedies and risk of hemorrhagic stroke. Neurology 68: 146–149. doi: 10.1212/01.wnl.0000250351.38999.f2 17210897 10.1212/01.wnl.0000250351.38999.f2

